# Protist-Type Lysozymes of the Nematode *Caenorhabditis elegans* Contribute to Resistance against Pathogenic *Bacillus thuringiensis*

**DOI:** 10.1371/journal.pone.0024619

**Published:** 2011-09-08

**Authors:** Claudia Boehnisch, Daniel Wong, Michael Habig, Kerstin Isermann, Nicolaas K. Michiels, Thomas Roeder, Robin C. May, Hinrich Schulenburg

**Affiliations:** 1 Institute for Evolution and Biodiversity, University of Muenster, Muenster, Germany; 2 Department of Animal Evolutionary Ecology, University of Tuebingen, Tuebingen, Germany; 3 School of Biosciences, University of Birmingham, Birmingham, United Kingdom; 4 Centre d'Immunologie de Marseille-Luminy, Université de la Méditerranée, Marseille, France; 5 INSERM, Marseille, France; 6 CNRS, Marseille, France; 7 Wellcome Trust Centre for Human Genetics, University of Oxford, Oxford, United Kingdom; 8 Department of Evolutionary Ecology and Genetics, University of Kiel, Kiel, Germany; 9 Department of Zoophysiology, University of Kiel, Kiel, Germany; Université de Genève, Switzerland

## Abstract

Pathogens represent a universal threat to other living organisms. Most organisms express antimicrobial proteins and peptides, such as lysozymes, as a protection against these challenges. The nematode *Caenorhabditis elegans* harbours 15 phylogenetically diverse lysozyme genes, belonging to two distinct types, the protist- or Entamoeba-type (*lys* genes) and the invertebrate-type (*ilys* genes) lysozymes. In the present study we characterized the role of several protist-type lysozyme genes in defence against a nematocidal strain of the Gram-positive bacterium *Bacillus thuringiensis*. Based on microarray and subsequent qRT-PCR gene expression analysis, we identified protist-type lysozyme genes as one of the differentially transcribed gene classes after infection. A functional genetic analysis was performed for three of these genes, each belonging to a distinct evolutionary lineage within the protist-type lysozymes (*lys-2*, *lys-5*, and *lys-7*). Their knock-out led to decreased pathogen resistance in all three cases, while an increase in resistance was observed when two out of three tested genes were overexpressed in transgenic lines (*lys-5*, *lys-7*, but not *lys-2*). We conclude that the lysozyme genes *lys-5*, *lys-7*, and possibly *lys-2* contribute to resistance against *B. thuringiensis*, thus highlighting the particular role of lysozymes in the nematode's defence against pathogens.

## Introduction

Lysozymes are small enzymes, which can cleave peptidoglycan, an essential component of bacterial cell walls. They are found in almost all groups of organisms and play important roles in both immunity and digestion [1]–[6]. In several organisms including the *Caenorhabditis* taxon the evolution of lysozymes is characterized by gene duplication and adaptive sequence evolution, leading to substantial intra-specific enzyme diversification [7]–[10]. *C. elegans* is of particular interest in this context, because its genome contains 15 phylogenetically diverse lysozyme genes, the largest number recorded to date [10].

*C. elegans* lysozymes are of two distinct types, the invertebrate- (*ilys* genes) and the protist- or Entamoeba-type (*lys* genes). The latter group further diverges into two distinct clades [10]. Representative members of the two main types are known from other organisms to act as functional antimicrobial enzymes [11], [12], suggesting that they have a similar function in *C. elegans*. In the nematode, the encountered genetic diversity may reflect functional diversity [10], in a similar way to that demonstrated for the antimicrobial *nlp* genes [13]. To date, only little information is available on the exact function of lysozymes in *C. elegans* immunity. All available data is based on genetic analysis, whereas none of the lysozymes have been characterized at the protein level. In particular, four lysozyme genes were directly shown by overexpression and mutant or RNAi-knock down analysis to contribute to the nematode's defence against pathogens: *lys-1* against *Serratia marcescens* and *Staphylococcus aureus* [14], [15]; *lys-2* against *Pseudomonas aeruginosa* [16]; *lys-7* against *Microbacterium nematophilum*, *P. aeruginosa*, *Salmonella* Typhimurium, the pathogenic *Escherichia coli* strain LF82, and *Cryptococcus neoformans* [16]–[20], and *ilys-3* against *M. nematophilum* [17]. In addition, seven other lysozymes have been implicated in immunity because exposure of *C. elegans* to various pathogens leads to changes in their transcription patterns [10], [21]–[25].

In the present study we focused on the role of protist-type lysozymes in *C. elegans* defence against the Gram-positive bacterium *B. thuringiensis* (Bt). Bt infects invertebrate hosts in a highly specialized process. The bacterium's infectious stages are spores associated with crystal toxins (Cry and Cyt toxins). After oral uptake of the spore-toxin mixture by a suitable host organism such as insects or nematodes, toxin solubilisation occurs inside the gut. The solubilised toxins specifically bind to glycolipids of intestinal cells [26]–[29], followed by formation of membrane pores and subsequent cellular disintegration [30]. Cell destruction appears to lead to a change in milieu (e.g., change of gut pH in insects) that triggers germination of spores and vegetative proliferation of bacteria [30], [31]. Most Bt strains express several different toxin genes [31], [32]. Overall, Bt produces an enormously diverse array of toxins and hence the taxon includes strains with high specificity towards different hosts including free-living nematodes such as *C. elegans* [33]–[35]. The nematode-specific Bt strains can establish persistent infections in *C. elegans* under laboratory conditions, even if the environmental medium does not support bacterial growth [33]–[36]. Some Bt strains are able to produce highly specific interactions with different natural *C. elegans* isolates [37], [38], suggesting that the two coexist in nature.

Previous studies characterized in much detail the nematode's defence against one of the nematocidal Bt toxins, namely Cry5B. The toxin binds to glycolipids on membranes of the epithelial cells in the intestine. Thus, alteration of these glycolipids and competitive binding of galectins to these glycolipids mediates resistance [26]–[29], [39], [40]. Resistance is also influenced by plasma membrane repair, as mediated by RAB-5- and RAB-11-dependent vesicle trafficking pathways [41]. Moreover, whole-genome microarray-based gene expression analyses and a recent RNAi knock-down screen revealed the involvement of a regulatory defence network based on two mitogen-activated protein kinase (MAPK) pathways, namely the p38 and JNK-like MAPK, and the activating protein 1 (AP-1) transcription factor [42], [43]. Protection against Bt toxins is additionally influenced by the hypoxia response and the insulin-like signaling pathways [44], [45]. The latter pathway also mediates both physiological defence and behavioural avoidance of a toxin-spore mixture of the pathogenic Bt strain B-18247 [46].

Here, we used microarrays to identify candidate immune effector genes in the *C. elegans* response against the nematocidal Bt strain B-18247, which is known to possess several different toxin genes [47]–[49]. Differential gene transcription was studied in three different natural *C. elegans* isolates (N2, MY15, and MY18), which show similar resistance to B-18247 (unpublished data), but have distinct genetic backgrounds [50], thus allowing identification of common principles in the genetic basis of resistance. As our transcriptional analysis identified a comparatively large number of protist-type lysozymes to be differentially regulated, we specifically tested the role of three lysozyme genes (*lys-2*, *lys-5*, *lys-7*) using knock-out mutants and gene overexpression in transgenic strains. The results strongly suggest a function of these lysozyme genes, especially of *lys-5* and *lys-7*, in the nematode's defence against pathogenic Bt.

## Results

### The transcriptional signature of Bt infected *C. elegans* reveals differentially regulated protist-type lysozymes

We explored the transcriptional response of three *C. elegans* strains (N2 and the two natural isolates MY15 and MY18) after 8 h exposure to the infectious spore-toxin mixture of the nematocidal Bt strain B-18247. The 8 h time point was specifically chosen, because it is sufficiently long after first pathogen exposure for an immune-relevant transcriptional response to develop [51], including the response against Bt toxin [43], and because it is well before the occurrence of Bt-induced host mortalities (>12 h after first exposure, unpublished data), which themselves associate with substantial transcriptional changes and thus could seriously complicate interpretation of inducible gene expression analysis [52].

Based on statistical analysis using F tests implemented in the R/MAANOVA package [53], [54], we obtained a list of significantly differentially transcribed genes. Although the three strains vary in their response, there is also substantial overlap in the differentially regulated individual genes (Table S1 and Table S2 in supporting information). Since transcription was studied in three genotypically different *C. elegans* strains, the overlapping gene set most likely represents the core set of genes that is inducible by the infectious Bt spore-toxin mixture. One of the prominent gene classes within this gene set are the protist-type lysozymes. Interestingly, some of them were consistently up- and others were consistently downregulated after exposure to the Bt spore-toxin mixture. In detail, significant upregulation was found for *lys-1* and *lys-2* in all three *C. elegans* strains and, additionally, for *lys-3* in the two recent natural isolates MY15 and MY18 (Table S1, Table S2). Significant downregulation was inferred for *lys-4* and *lys-5* in all three strains, for *lys-10* in strains MY15 and MY18, and for *lys-7* in MY18 (Table S1, Table S2).

**Figure 1:**
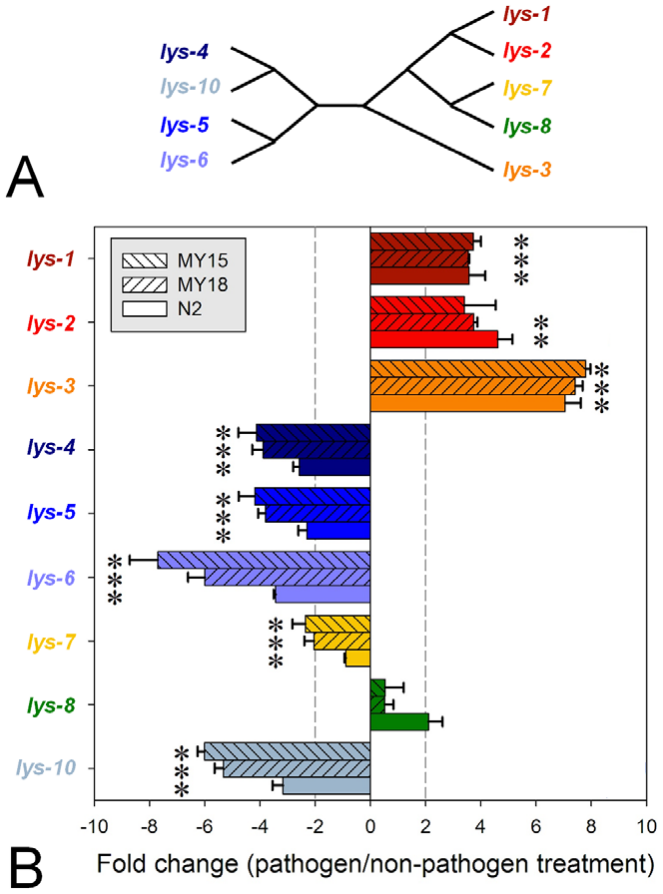
Information on lysozymes including their phylogenetic relationships and Bt-induced expression profiles. The phylogeny shown in (A) is derived from previously published phylogenetic analysis [10]. Expression profiles are given in (B) and were established from quantitative realtime PCR. The three *C. elegans* strains (MY15, MY18, N2) were confronted either with pathogenic or non-pathogenic Bt for 8 h. Lysozyme induction is given as the normalized expression difference between pathogen and non-pathogen treatment, such that positive values indicate upregulation and negative values downregulation after pathogen exposure. Relative expression levels were calculated from crossing points following the 2^−ΔΔCT^ method (see methods section). Reddish/yellowish bar colours refer to the lysozymes from chromosome V (*lys-1*, *lys-2*, *lys-3*, and *lys-7*), bluish colours to those from chromosome IV (*lys-4*, *lys-5*, *lys-6*, and *lys-10*), and green to that from chromosome II (*lys-8*). An expression difference of 2 or −2 is indicated by dashed vertical lines. Stars highlight groups that are significantly different from 0 according to a t test and false-discovery-rate adjusted significance levels (all groups with the exception of the three *lys-8* groups and *lys-2* from MY15).

Differential gene expression of the protist-type lysozymes was subsequently reassessed using quantitative reverse transcription real-time PCR (qRT-PCR). The qRT-PCR fold induction values were calculated with the 2^−ΔΔCT^ method [55] and clearly differed among genes (Figure 1). More importantly, they generally validated the microarray results (Table S1, Table S2, Figure 1). The exceptions included significant upregulation of *lys-3* in N2, and significant downregulation of *lys-7* in MY18 and N2, and *lys-10* in N2, which we consistently inferred by qRT-PCR (Figure 1) but not by microarray analysis. Note that even though the microarrays contained oligos for almost all protein-coding genes of the *C. elegans* genome (20,334 genes), unfortunately the *lys-6* gene was not included. Thus, for this gene we only have results from qRT-PCR analysis. Taken together, our results suggest that exposure to the Bt spore-toxin mixture leads to upregulation of three closely related lysozyme genes and, at the same time, downregulation of five other lysozymes, of which four belong to the same evolutionary lineage (Figure 1), possibly indicating functional divergence of the lysozymes across the genealogical tree.

### Protist-type lysozymes influence resistance against Bt

**Figure 2:**
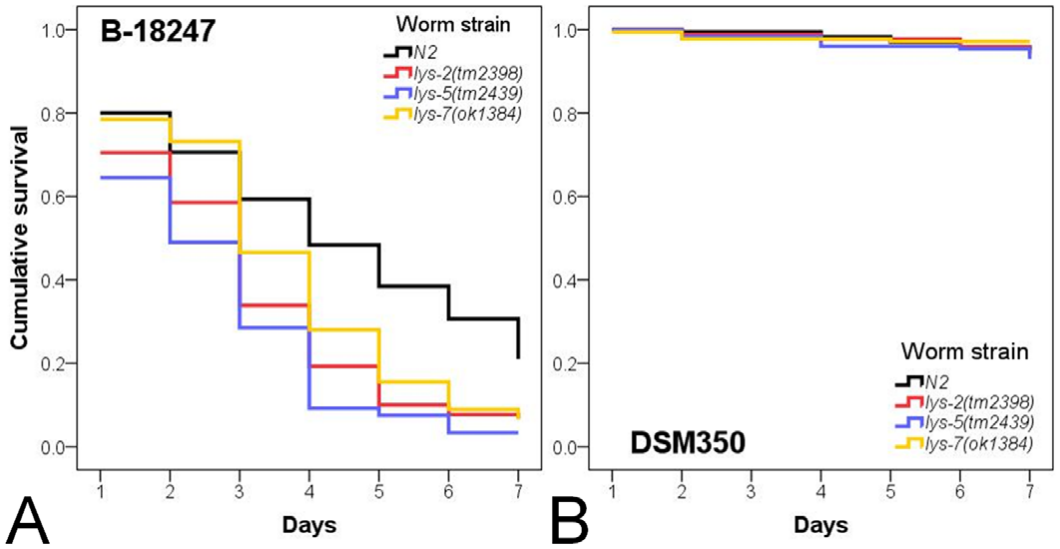
Survival of *C. elegans* knock-out strains. Survival rate was studied on (A) the nematocidal Bt strain B-18247 or (B) the non-nematocidal Bt strain DSM-350. It was checked daily for a period of 7 days. Every other day worms were transferred to fresh treatment plates. The *C. elegans* wildtype N2 was compared to the mutants with lysozyme knock-out alleles *lys-2(tm2398)*, *lys-5(tm2439)*, and *lys-7*(*ok1384*).

**Figure 3:**
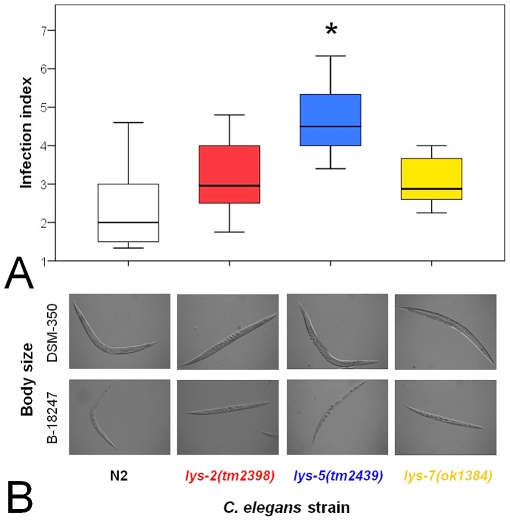
Variation among knock-out strains in infection load and body size. Infection load (A) and body size (B) were compared after 8 h exposure among the wildtype N2 and the mutants with lysozyme knock-out alleles *lys-2(tm2398)*, *lys-5(tm2439)*, and *lys-7*(*ok1384*). Nematodes were confronted with the pathogenic Bt strain B-18247 (panel A) or both the pathogenic strain and the non-pathogenic strain DSM-350 (panel B). The results for infection load are shown as box–plots, where the horizontal black line gives the median and the boxes the interquartile range (25% of the data above and below the median). Nomarski images show examples of body sizes for the different strains and treatments. Detailed statistics are given in Table S3.

**Figure 4:**
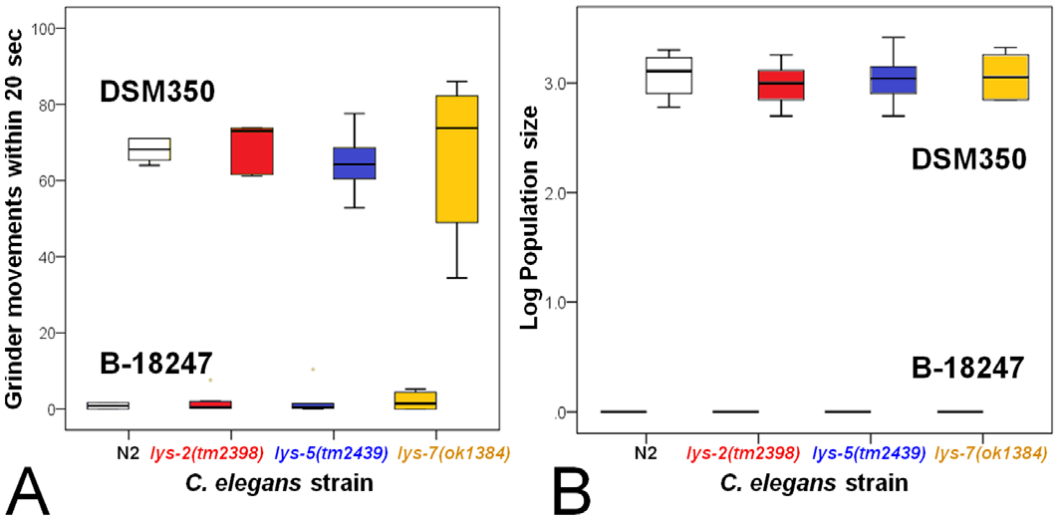
Variation among knock-out strains in feeding rate and population size. Feeding rate (A) and population size (B) were studied for the wildtype N2 and the mutants with lysozyme knock-out alleles *lys-2(tm2398)*, *lys-5(tm2439)*, and *lys-7*(*ok1384*). Nematodes were confronted with either the pathogenic Bt strain B-18247 (results are found in the bottom part of each panel) or the non-pathogenic strain DSM-350 (top part of each panel). Feeding rate was determined by counting grinder movements within a 20 sec period after 8 h exposure. Population size assays were initiated with ten L4 larvae and the total number of worms were scored after five days. Results are shown as box–plots, where the horizontal black line gives the median and the boxes the interquartile range (25% of the data above and below the median). Population size is shown in logarithmic scale.

We tested the idea of functional diversification using phenotypic analysis of Bt infected *C. elegans* knock-out mutants and transgenic worms overexpressing lysozyme genes. In particular, the resistance function of three lysozyme genes was evaluated, belonging to the three distinct protist-type lysozyme clades (Figure 1) and including one of the upregulated genes (*lys-2*) and two of the downregulated genes (*lys-5* and *lys-7*). Phenotypic effects on pathogen defence were determined by measuring survival rate, infection load, body size, feeding rate, and population size. In the presence of pathogenic Bt, all three knock-out mutants showed significantly decreased survival when compared to the wild-type N2 (Figure 2, Table S3 in supporting information). Moreover, the *lys-5(tm2439)* mutant also suffered from significantly higher infection load than N2 (Figure 3, Table S3), whereas the other two mutants did not vary significantly from the wild-type. On pathogenic Bt, none of the mutants differed significantly from the wild-type N2 regarding body size, feeding rate, and population size (Figure 3, Figure 4, Table S3). We did not observe any significant differences among strains on the non-pathogenic Bt control (Figure 2, Figure 3, Figure 4).

**Figure 5:**
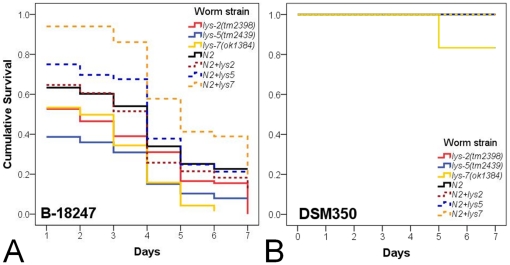
Survival of knock-out strains and corresponding transgenic lines with lysozyme overexpression. Survival was evaluated in the presence of (A) the nematocidal Bt strain B-18247 or (B) the non-nematocidal Bt strain DSM-350. Survival was checked daily for a period of 7 days. Every other day worms were transferred to fresh treatment plates. The transgenic lines overexpressed either *lys-2*, *lys-5*, or *lys-7* in the N2 genomic background. The knock-out mutants had the following alleles: *lys-2(tm2398)*, *lys-5(tm2439)*, *lys-7*(*ok1384*).

We additionally tested lysozyme function by overexpressing the three genes in the N2 wild-type background. When exposed to the pathogenic Bt strain B-18247, overexpression of *lys-7* led to significantly higher survival rates than observed for the corresponding *lys-7* knock-out mutant and also the N2 wild-type (Figure 5, Table S4 in supporting information). Overexpression of *lys-5* resulted in significantly increased survival only relative to the corresponding *lys-5* knock-out mutant but not N2, whereas *lys-2* overexpression did not differ in survival to the corresponding knock-out or N2 (Figure 5, Table S4). There was no significant variation among strains exposed to the non-pathogenic Bt control (Figure 5).

## Discussion

In the present study we employed whole genome microarrays to explore the involvement of candidate immune effectors in the nematode's inducible defence against a nematocidal strain of the Gram-positive bacterium *Bacillus thuringiensis*. During this analysis, we specifically controlled three critical but often neglected variables: (i) Rather than using the standard food source *E. coli* as a control, we exposed nematodes to the spore-toxin mixture of a Bt strain (DSM-350) that does not infect nematode host tissue or reduce *C. elegans* survival [36], [46]. (ii) We always added *E. coli* as an *ad libitum* food source to both pathogen and control treatments. These first two points served to ensure that the observed inducible transcriptional response is specific to the nematocidal pathogenicity of the bacterium and is unlikely to be compromised by taxonomic differences between test and control bacterial species or the availability of food. (iii) We focused on differential transcription after 8 h exposure – before first pathogen-induced mortalities occur (usually not before 12 h after exposure', unpublished results), which could bias transcriptional inferences [52], and, at the same time, after inducible defence responses should have had the time to develop [51], including those against Bt toxin [43].

Our transcriptome analysis identified lysozyme genes to be among the differentially transcribed gene classes, suggesting a role in pathogen defence. For a more detailed analysis, we focused on the protist-type lysozymes. Interestingly, the groups of up- versus down-regulated genes fall into distinct evolutionary lineages (Figure 1), possibly suggesting an evolutionary differentiation of gene function upon gene duplication as previously proposed [10]. In particular, we expected a role of the up-regulated lysozyme genes in immune defence against pathogenic Bt, whereas the downregulated lysozymes should not be required under these conditions but under non-pathogenic conditions may have a different function, for example as digestive enzymes [10]. In our study, both treatments included a suitable food source (*E. coli*). If the presence of pathogens is speculated to inhibit expression of digestive enzymes, then their absence under control conditions and the simultaneous presence of food could have elicited a digestive response. Under these assumptions, the lysozyme genes of particular importance for digestion could appear as a downregulated gene set under pathogen conditions even if they are of no relevance to the nematode's response to pathogens.

The results from our functional genetic analysis disagree with our expectations. This analysis specifically focused on three genes (*lys-2*, *lys-5*, *lys-7*) as representatives of the up- as well as down-regulated gene classes and also of the three distinct evolutionary lineages of the protist-type lysozyme genealogy [10]. Manipulation of these three genes consistently produced similar phenotypes: Knock-out mutations resulted in significantly reduced resistance measures during exposure to pathogenic Bt, whereas the overexpression of at least *lys-5* and *lys-7* consistently led to significantly higher survival rates on the pathogen (Figures 2, 3, 4, 5). At the same time, strains did not vary significantly on the non-pathogenic Bt control, indicating that the observed variation on the pathogen is unlikely due to a general deficiency of the mutants (Figures 2, 3, 4, 5). Moreover, the KO mutants also did not vary significantly in feeding behaviour on the pathogen (Figure 4), which argues against a role of the mutations in behavioural defence such as the pathogen-induced reduction in ingestion [56]. Consequently, the observed variation on the pathogen strongly suggests a function of the tested lysozymes in physiological immunity. Our findings then also suggest that pathogen-dependent gene expression patterns are not necessarily indicative of a gene's role in defence, especially in case of gene downregulation after pathogen exposure. Defence genes may be present in the downregulated gene set if the pathogen directly interferes with its expression as part of its attempt to compromise host resistance and thus facilitate invasion. Such interference with the host's immune system is known for a large variety of pathogen taxa [57], including *P. aeruginosa* in a *C. elegans* host model [58]. Another non-exclusive explanation may be that the downregulated lysozymes are part of the constitutively expressed (rather than inducible) immune system and that their transcription is decreased upon pathogen-mediated damage.

Based on our analysis, especially the functional genetic approach, we conclude that at least the lysozyme genes *lys-5* and *lys-7* contribute to physiological immunity against *B. thuringiensis*. For *lys-2*, a resistance function is indicated by the knock-out analysis, whereas its overexpression does not lead to a change in phenotype. The latter finding may suggest that endogenous expression levels for this gene (as shown by the wild-type N2) are sufficient for maximal resistance to Bt-induced killing. Taken together, our study adds to our understanding of the putative immune effector repertoire of *C. elegans* and the particular role of lysozymes in this context. Here, it is of interest that none of the previous genetic analyses obtained an indication for a defence function of *lys-5* [10]. A possible reason is that most studies based on a microarray-transcriptome approach for identification of candidate immunity genes focus on the upregulated gene sets. If *lys-5* generally tends to be downregulated in response to pathogen exposure, as in our study, then its role in defence would not have been evaluated using functional genetic analysis. In fact, *lys-5* transcription was previously found to be decreased in response to pathogenic *P. aeruginosa*, but in this case the gene was not included in the subsequent genetic analysis [51]. For *lys-2* and *lys-7*, a function in defence against *B. thuringiensis* was previously unknown. At the same time, these genes, especially *lys-7*, were implicated in resistance against other pathogens [16]–[20], suggesting that either both of them or at least *lys-7* play a more central role in *C. elegans* immunity.

We would like to emphasize that our study (and most previous studies) only indicates, but does not strictly prove a defence function of these enzymes. Unequivocal evidence would require analysis of the purified protein, especially its ability to interact with the pathogen at the molecular level. In fact, such unequivocal evidence is as yet only available for a single *C. elegans* immune effector, namely the saposin-like caenopore SPP-5. For this caenopore, it was possible to solve its tertiary structure and, most importantly, its ability to form pores into bacterial cell membranes [59], [60]. Such biochemical studies remain a particular but necessary challenge for an exact understanding of *C. elegans* immune effectors.

## Methods

### Nematode and bacterial strains, general conditions of experiments

The *C. elegans* strain N2 and two natural isolates from Germany, MY15 and MY18 [50], were used to study transcriptional variation. The function of three lysozyme genes was investigated using knock-out (KO) mutants (*lys-2(tm2398)*, *lys-5(tm2439)*, and *lys-7*(*ok1384)*), produced by the Japanese National Bioresource Project for the experimental animal “nematode *C. elegans*”, coordinated by the Shohei Mitani laboratory (Tokyo Women's Medical College, Tokyo, Japan), and the American *C. elegans* gene knockout consortium (Oklahoma Medical Research Foundation). We confirmed that the mutants are homozygous for the respective deletions by gene-specific PCRs (see allele information at Wormbase, www.wormbase.org). Mutants were backcrossed five times to N2 and did not show any apparent phenotypic aberrations under standard laboratory conditions. Strains are available from the Caenorhabditis Genetics Center (www.cbs.umn.edu/CGC/).

Independent transgenic lines were constructed for *lys-2*, *lys-5* and *lys-7* in the N2 background: MY1021 (wt; *yaEx16*(*lys-2::gfp; pmyo-2::rfp*)), MY1022 (wt; *yaEx17*(*lys-5::gfp; pmyo-2::rfp*)), and MY1017 (wt; *yaEx12*(*lys-7::gfp; pmyo-2::rfp*)). The lines were generated following the PCR fusion approach [61], in each case including the lysozyme gene of interest and its 5′-upstream region (5′ upstream regions: 735 bp for *lys-2*, 1422 bp for *lys-5*, and 708 bp for *lys-7*), amplified with the following primers: Lys-2_for (5′-taaatatttccgatgtgattgtcg -3′), Lys-2_nest (5′-tgattgtcgataacctttttaacg-3′), Lys-2_gfprev (5′- agtcgacctgcaggcatgcaagctgtttccgacaaatcctccgacaacaa-3′), Lys-5_for (5′-aagtcacaacatgtggtagctgat-3′), Lys-5_nest (5′-ttcacagttatgaattctgcgttt-3′), Lys-5_gfprev (5′-agtcgacctgcaggcatgcaagcttggaatgtagttcatatcaac -3′), Lys-7_for (5′-gactttggtgcttaggaaagatg-3′), Lys-7_nest (5′-tagtattcagaacgtggcggttag-3′), Lys-7_gfprev (5′- agtcgacctgcaggcatgcaagctaattttcagacttccttgcacaaat -3′). Germline transformation followed the standard approach [62], using 5 ng/µl of the transgene and 30 ng/µl of the *pmyo-2::rfp* co-injection marker. The resulting transgenic lines did not show any phenotypic aberrations under standard laboratory conditions.

The pathogenic Bt strain NRRL B-18247 was obtained from the Agricultural Research Service Patent Culture Collection (United States Department of Agriculture, Peoria, IL, USA) and the non-pathogenic strain DSM-350 from the German Collection of Microorganisms and Cell Cultures (Deutsche Sammlung von Mikroorganismen und Zellkulturen GmbH, Braunschweig, Germany). A spore-toxin mixture was prepared for each strain as previously described [36], [63], aliquotted and stored at −20°C, at which they preserve their activity for approximately a year (unpublished data) [64]. Aliquots were freshly thawed before usage in the different experiments.

Nematodes were maintained on nematode growth medium (NGM) plates inoculated with *E. coli* OP50 following standard protocols [65]. The experiments were carried out at 20°C with hermaphroditic fourth instar larvae (L4) that were either exposed to the pathogenic or the non-pathogenic Bt strain. *E. coli* strain OP50 was always added *ad libitum* as an independent food source. Peptone free medium (PF) was used instead of NGM in order to prevent germination of the spores [46].

### Pathogen exposure and RNA isolation

Cultures of the three nematode strains (MY15, MY18 and N2) were synchronised developmentally by sodium hypochlorite treatment [65]. Ca. 20,000 L4 nematodes were transferred to PF plates (14.5 cm diameter), containing 2.86 ml of a 10∶1 mixture of *E. coli* OP50 and Bt in PBS buffer (either the pathogenic B-18247 or the control DSM-350 strain; final Bt concentration of 1.5×10^8^ spores/ml). Each nematode strain was exposed for 8 h to either pathogenic or non-pathogenic Bt in either three (strain N2) or four independent replicates (strains MY15 and MY18). Thereafter, nematodes were washed off, pelleted by centrifugation, washed in 10 ml sterile Millipore H_2_O to remove external bacteria, pelleted, snap-frozen in liquid nitrogen, followed by addition of 1.5 ml Trizol (Invitrogen) on ice (4°C), RNA extraction according to manufacturer's instructions (Trizol, Invitrogen), and storage of RNA isolates at −20°C.

### Microarray analysis

Gene expression patterns were compared between pathogenic and non-pathogenic treatments for the three *C. elegans* strains separately (MY15, MY18, N2) using whole genome microarrays containing oligonucleotide sequences of 20,334 genes, designed at the Genome Sequencing Center (St. Louis, USA) and spotted on UltraGAPS™ slides (Corning) at the Plateforme Transcriptome (Marseille-Nice génopole/CNRS/INRA; Sophia Antipolis, France). 10 µg total RNA was employed for cDNA synthesis with SuperScript II Reverse Transcriptase (SSII, Invitrogen) using oligo dT_24_-V primer (Sigma-Aldrich) and aminoallyl-dUTP (Sigma-Aldrich) nucleotide analogs. cDNA was purified with the Qiagen PCR purification kit and labelled either with Cy 3 or Cy 5 mono-reactive dye packs (Amersham). Two differentially labelled probes were hybridized to the microarray slides at 45°C for 16 h in hybridization buffer (1X SSC, 0.2% SDS, 7 mM Tris-Cl, 0.2 mg/mL yeast t-RNA (Invitrogen), 0.2 mg/mL poly(A)DNA (Sigma-Aldrich)) Fluorescent images were captured using ScanArray 4000XL (Perkin Elmer) and quantified with the software QuantArray version 2.1 (Perkin Elmer). Cy 3/Cy 5 dyes were swapped between Bt treatments across the independent replicates. The microarray data is described in accordance with MIAME guidelines and deposited at ArrayExpress (accession number E-MEXP-2168; http://www.ebi.ac.uk/microarray-as/ae/).

Microarray signal intensities were normalized in three steps. We first subtracted average background fluorescence for each spot. Thereafter, data were transformed with the programme R/MAANOVA [54], using first the rlowess and then the linlog transformation [54], [66]. Differential gene expression was assessed with a mixed regression model, including pathogen as a fixed and array as a random factor using the restricted maximum likelihood (REML) approach [53], [54], [67]. The pathogen effect was evaluated with an F_3_-test using a pooled estimator of the error-variance [53], [54] and comparison of the tabulated p-values with the F distribution rather than a permutation analysis, which was unsuitable for our study because of low sample size (maximum of four replicates). To correct for multiple testing we adjusted the significance level with the help of the false discovery rate (FDR) [68].

### Quantitative Realtime-PCR (RT-PCR)

Microarray-based transcription changes were specifically evaluated for nine protist-type lysozyme genes with quantitative reverse transcriptase Realtime PCR (RT-PCR). The expression patterns were compared between pathogenic and control treatments for each strain (MY15, MY18, N2) and, in each case, three independent replicates. The lysozymes were amplified with gene-specific primers (Table 1). Their expression was normalized by comparison with expression of the elongation factor 1α-homologue *K07A12.4* (EF) reference gene.

**Table 1:** Primer used for lysozyme qRT-PCR gene expression analysis.

Name	Oligo-Sequence [5′ - 3′]	Gene
lys-1f2	GAACTGCCTCAAGACATCCA	*lys-1*
lys-1r2	CCAATCCAGCAGAATAAGCA	
lys-2r2	GACGTTGGCAGTTGGATTG	*lys-2*
lys-2f2	GCTGGATTGGGAATTGAGAC	
lys-3r2	TGGGGAGTTTCGTTCATCATA	*lys-3*
lys-3f2	CAGCTCCTCTTCAAACGAATG	
lys-4f4	AGGCAATGGTCAGAGAAGCTC	*lys-4*
lys-4r4	TGAGAAATCCTTAATTCCATCATAG	
lys-5f1	CAGAATTTATCATTCATCGGG	*lys-5*
lys-5r1	TCAAGCCATAGAGTGGAGATC	
lys-6r4	ACTGCATCAAGAGACGCCTTA	*lys-6*
lys-6f4	TCAGAATGTGGCCAACGCA	
lys-7f3	GTCTCCAGAGCCAGACAATCC	*lys-7*
lys-7r3	CCAGTGACTCCACCGCTGTA	
lys-8f	GCTTCAGTCTCCGTCAAGGTC	*lys-8*
lys-8r	TGAAGCTGGCTCAATGAAAC	
lys-10f3	GGTTAAAGAAGCCGAGGCTAGA	*lys-10*
lys-10r3	TTCCATCCGCCGAAAGCTA	
EF1	CAGGATTTGAAAACGGAGGA	*K07A12.4*
EF2	AAAGCCAGCCTGACGAGTTTA	

For the strains MY15 and MY18, cDNA was synthesized at 42°C for 1 h using 5 µg total RNA, 1× first strand buffer, 10 mM DTT, 1 mM dNTP, 1 µM Capfinder primer CFB1-rG (5′- GAGAGAACGCGTGACGAGAGAGACTGACArGrGrG -3′), 1 µM dT-T7 Primer, 0.8 mM MnCl_2_, 1 U RNase inhibitor, 10 U Superscript III (Invitrogen), followed by reaction termination at 65°C for 10 min. RT-PCR was carried out with the Epicentre Biotechnologies TAQurate™ GREEN Real-Time PCR Mastermix kit and the LightCycler System, using for each RT-PCR run and primer pair two replicates of non-template controls and two replicates of template samples based on the following reaction conditions: initial denaturation for 135 sec at 95°C, followed by 35 cycles of 5 sec at 94°C, 15 sec at 60°C and 15 sec at 72°C (temperature transition rate of 20°C/sec). Amplification was followed by a melting-temperature identification cycle in order to assess PCR product purity (10 sec at 95°C, cooling to 60°C (temperature transition rate of 20°C/sec), followed by slow heating to 95°C (temperature transition rate of 0.1°C/sec)).

For N2, RT-PCR was performed by the Microarray Facility of Tuebingen University, Germany, using the LightCycler System 480 (Roche Diagnostics). cDNA was synthesized with the QuantiTect cDNA Synthesis Kit (Qiagen) using 0.5 µg RNA an incubation for 15 min at 42°C in 1× RT buffer, 1 µM per primer mix (oligo-dT plus random hexamer primer), and reverse transcriptase, followed by reaction termination at 95°C for 5 min. RT-PCR generally followed the above protocol using a 1∶10 cDNA dilution and analysis in 384-well plate format. For each primer pair, one replicate of a non-template control and three replicates of template samples were prepared, containing 5 µl 2× QuantiTect SYBR Green Mix, 300 µM forward and reverse primer, and either 2 µl cDNA (equivalent to 5 ng total RNA) or no cDNA (non-template control). The reaction consisted of initial denaturation for 15 min at 95°C, followed by 45 cycles of 45 sec at 95°C, 20 sec at 55°C, and 15 sec at 72°C. After amplification the melting curve was recorded as above.

Expression data was obtained as C_T_ values, which corresponds to the cycle number of the amplification reaction, at which the fluorescence of the sample exceeds the background level for the first time, and which were measured within the linear amplification ranges. Linear regression was used for baseline correction of each sample as implemented in the program LinRegPCR (http://LinRegPCR.nl) [69], [70]. Analysis of relative gene expression followed the comparative 2^−ΔΔCT^ method [55]. Transcription differences larger than 2 or smaller than −2 were then taken as an indication for significant differential gene expression. A significant difference from 0 was additionally evaluated with t tests and an adjustment of significance levels according to FDR. The statistical analyses were performed with the programme JMP 8.0 (SAS Inst. Inc.) and the graphical summary was produced with SigmaPlot 11.0 (Systat Software Inc.).

### Functional analysis of selected lysozyme genes

We investigated the role of the protist-type lysozymes *lys-2*, *lys-5*,and *lys-7* in defense against pathogenic Bt by gene knock-out and transgenic lines with gene overexpression, followed by phenotypic analysis. Resistance was evaluated as survival rate and infection load. We furthermore assessed nematode body size, feeding rate and population size, the latter being a compound fitness measure determined by reproductive rate and developmental time that indicates the pace at which worms could colonize a new habitat. Population size and body size assays were performed in 5 cm (diameter) “wormballs” containing PF medium [46], [71]. The wormballs were inoculated with 700 µl bacterial suspensions (350 µl per half) containing *E. coli* OP50, and - in a 1∶10 dilution - either Bt18247 or DSM350 (final Bt concentration of 1.5×10^9^ spores/ml). All other assays were performed in 3 cm petri dishes containing PF medium.

The survival rate of the KO mutants was examined using a total of 20 replicates per treatment combination (two runs with ten replicates). The survival assays for the transgenic worms were conducted on three different dates with five replicates per run yielding 15 replicates in total, with the exception of MY1022 and all strains on the non-pathogenic control, for which ten replicates were assayed. Ten L4 hermaphrodites per worm strain were transferred manually to each plate. Survival rate of the worms was checked daily for seven days by recording the number of alive worms, dead worms and lost worms (e.g., dead worms on the edge of the agar plate, lost worms on day of transfer or alive worms on day 7). Worms were transferred to fresh plates every other day.

Body size was measured after 8 h Bt exposure (either control or nematocidal strain) in two independent runs with five replicates per run and treatment combination. Infection load was determined for three to five surviving worms exposed to pathogenic Bt for 8 h using five replicates per strain. For body size and infection load measurements, worms were transferred into a drop of M9 buffer on a diagnostic microscope slide, frozen at −20°C, and then photographed with a LeicaDFC 320 camera (Leica Microsystems Imaging solutions Ltd, Cambridge, UK) attached to a Leica DM 5000 B microscope (Leica Microsystems, Wetzlar, Germany), using Normarski settings and 10× magnification for body size and 100× magnification for infection. Body size was measured as the two-dimensional area of an animal using the program ImageJ [72], followed by calculation of the average body size for each replicate. The infection level was inferred from surviving worms using a similar approach as previously described [46] based on seven infection categories: (i) no infection; (ii) no more than 20 spores in the gut; (iii) more than 20 and no more than 100 spores, accumulation in either the anterior or posterior part of the gut, no vegetative cells; (iv) as category iii, but accumulations in both anterior and posterior part of the gut; (v) more than 100 spores with massive accumulations in the anterior and/or posterior part of the gut, first intestinal cells destroyed, no vegetative cells; (vi) as category v, but with vegetative cells inside the gut; (vii) as category vi but spores and vegetative cells in high concentrations in all parts of the gut and body. For each replicate, we calculated an overall infection index as the average from at least three and maximal five surviving worms. For body size and infection load analysis, we excluded replicates with data from less than three surviving worms.

Feeding rate was assessed on minimum agar (3.4% w/v), inoculated with 40 µl of a mixture of *E. coli* (1.5×10^10^ cells/ml) and Bt (either the nematicidal or the nonpathogenic BT; concentration in both cases, 3.67×10^7^ spores/ml). Ten L4 were transferred onto each plate. After 8 h, the feeding rate was determined for five individuals from within the bacterial lawn by counting pharynx grinder movements within a 30 s period [46]. Data was considered from 5 independent experiments, yielding 25 worms per factor combination. Nematode population size was examined in parallel to the above assays using a total of ten replicates per treatment combination (two runs with five replicates each). Ten L4 worms per strain were transferred manually to each wormball. After five days the total number of worms per population was determined.

Statistical differences in body size, infection load, feeding rate, and population size were separately tested for significance using pair-wise comparisons of KO mutants with corresponding N2 controls within each treatment, using rank-based Wilcoxon tests. Differences in infection load were evaluated with a One-Way-ANOVA, followed by Tukey HSD post-hoc tests. Differences in survival rate were evaluated with the Kaplan-Meier approach followed by log-rank tests. KO-mutants were always compared to the wildtype N2. Transgenic worms were compared to N2 and also the corresponding KO mutant strain. Significance levels were adjusted using FDR [68]. The statistical analysis was performed with the programme SPSS 16.0 (SPSS Inc.).

## Supporting Information

Table S1 **Lists of significantly differentially regulated genes upon exposure of three** ***C. elegans*** **strains to nematocidal** ***B. thuringiensis*** **B-18247.**
(XLS)

Table S2 **Summary of the significantly differentially regulated genes upon pathogen exposure of** ***C. elegans*** **.**
(XLS)

Table S3 **Phenotypes of lysozyme knock-out mutants.** Control treatment was performed with the non-nematocidal Bt DSM-350, the pathogen treatment with the nematocidal Bt strain B-18247. Phenotypic measures are given as means and, in brackets, standard errors. Survival rate was followed daily over 7 days (mean given as number of days of survival), infection load was determined after 8 h, and body size (multiplied by 10^2^ in mm^2^) was determined after 1 day, and population size after 5 days. After 8 h, the pumping rate was determined for five individuals by counting the pharynx grinder movements within a 30 s period. Infection load under control conditions was not determined (nd). Survival experiments were performed on two separate dates for a total of 20 replicates per treatment combination (10 replicates per date and treatment combination). For body size (10 replicates in total) and infection load (5 replicates in total), replicates were excluded if less than 3 worms were available for analysis. Survival rates were analysed using the Kaplan-Meier approach followed by a post-hoc log-rank test for comparisons between the KO mutants and N2. Differences in infection load were evaluated using One-Way-ANOVA followed by the post-hoc Tukey HSD test for pairwise comparisons between KO mutants and N2. Statistical analyses of differences in pumping rate, body size and population size were based on rank-based Wilcoxon test, p-values were adjusted using FDR to account for multiple testing. For all nematode strains, exposure to pathogenic Bt (factor Bt treatment) led to a significant decrease in survival rate, pumping rate, body size, and population size. Significant differences between N2 and the KO mutants under pathogenic conditions are shown in bold and indicated by *. The infection load of *lys-7(ok1386)* only showed a trend in being different to N2 (indicated by ^+^).
(DOCX)

Table S4 **Survival rate of lysozyme knock-out mutants and transgenic worms on pathogenic and non-pathogenic** ***B. thuringiensis*** **.** The pathogen treatment was performed with Bt strain B-18247. Survival measures are given as means and, in brackets, standard errors. Survival rate was followed daily over 7 days. The survival experiment on the pathogen was repeated on 3 separate occasions for a total of 15 replicates per strain. Only 10 replicates were assayed on the pathogen for N2+*lys-5* (i.e., overexpression of *lys-5* in N2 background) and all strains on the non-pathogenic control. Under control conditions, individuals of almost all strains survived until the end of the assay period of seven days. Survival rates were analysed using the Kaplan-Meier approach followed by a post-hoc Log-rank test for comparisons between the KO mutants and the corresponding transgenic strain as well as for comparisons between the transgenic strains and N2. Significant differences between transgenic strains and the corresponding KO mutant are shown in bold and indicated by *. Significant differences between N2 and transgenic strains are indicated with ^+^. Because of multiple testing we adjusted significance levels using FDR.
(DOCX)
