# Supplementary material for: Protist-Type Lysozymes of the Nematode Caenorhabditis elegans Contribute to Resistance against Pathogenic Bacillus thuringiensis
Source: PLoS One. 2011 Sep 8;6(9):e24619. doi: 10.1371/journal.pone.0024619 (PMC3169628; doi:10.1371/journal.pone.0024619)
Supplement: Table S3 — Phenotypes of lysozyme knock-out mutants. Control treatment was performed with the non-nematocidal Bt DSM-350, the pathogen treatment with the nematocidal Bt strain B-18247. Phenotypic measures are given as means and, in brackets, standard errors. Survival rate was followed daily over 7 days (mean given as number of days of survival), infection load was determined after 8 h, and body size (multiplied by 102 in mm2) was determined after 1 day, and population size after 5 days. After 8 h, the pumping rate was determined for five individuals by counting the pharynx grinder movements within a 30 s period. Infection load under control conditions was not determined (nd). Survival experiments were performed on two separate dates for a total of 20 replicates per treatment combination (10 replicates per date and treatment combination). For body size (10 replicates in total) and infection load (5 replicates in total), replicates were excluded if less than 3 worms were available for analysis. Survival rates were analysed using the Kaplan-Meier approach followed by a post-hoc log-rank test for comparisons between the KO mutants and N2. Differences in infection load were evaluated using One-Way-ANOVA followed by the post-hoc Tukey HSD test for pairwise comparisons between KO mutants and N2. Statistical analyses of differences in pumping rate, body size and population size were based on rank-based Wilcoxon test, p-values were adjusted using FDR to account for multiple testing. For all nematode strains, exposure to pathogenic Bt (factor Bt treatment) led to a significant decrease in survival rate, pumping rate, body size, and population size. Significant differences between N2 and the KO mutants under pathogenic conditions are shown in bold and indicated by *. The infection load of lys-7(ok1386) only showed a trend in being different to N2 (indicated by +). (DOCX) [file pone.0024619.s003.docx]

**Table S3. Phenotypes of lysozyme knock-out mutants.**

| **KO allele** | **Survival rate** | **Infection load** | **Body size** | **Feeding rate** | **Population size** |
| --- | --- | --- | --- | --- | --- |
| **Control treatment** |  |  |  |  |  |
| N2 wildtype | 6.89 (0.04) | nd | 7.43 (0.33) | 70.00 (3.09) | 1270 (156.38) |
| *lys-2(tm2398)* | 6.89 (0.05) | nd | 6.13 (0.55) | 74.72 (7.8) | 1040 (131.82) |
| *lys-5(tm2439)* | 6.84 (0.06) | nd | 7.18 (0.17) | 64.72 (4.14) | 1220 (193.68) |
| *lys-7(ok1386)* | 6.87 (0.06) | nd | 7.53 (0.10) | 65.08 (10.02) | 1192 (216.80) |
| **Pathogen treatment** |  |  |  |  |  |
| N2 wildtype | 4.27 (0.17) | 2.6 (0.34) | 3.34 (0.16) | 1.48 (0.93) | 20 (13.33) |
| *lys-2(tm2398)* | **3 (0.14)*** | 3.39 (0.29) | 3.41 (0.38) | 2.08 (1.42) | 0 (0) |
| *lys-5(tm2439)* | **2.62 (0.13)*** | **4.4 (0.19)*** | 2.87 (0.30) | 2.52 (1.98) | 0 (0) |
| *lys-7(ok1386)* | **3.51 (0.13)*** | 3.59 (0.24)^+^ | 2.87 (0.24) | 2.2 (1.1) | 20 (13.33) |
