# Supplementary material for: Protist-Type Lysozymes of the Nematode Caenorhabditis elegans Contribute to Resistance against Pathogenic Bacillus thuringiensis
Source: PLoS One. 2011 Sep 8;6(9):e24619. doi: 10.1371/journal.pone.0024619 (PMC3169628; doi:10.1371/journal.pone.0024619)
Supplement: Table S4 — Survival rate of lysozyme knock-out mutants and transgenic worms on pathogenic and non-pathogenic B. thuringiensis . The pathogen treatment was performed with Bt strain B-18247. Survival measures are given as means and, in brackets, standard errors. Survival rate was followed daily over 7 days. The survival experiment on the pathogen was repeated on 3 separate occasions for a total of 15 replicates per strain. Only 10 replicates were assayed on the pathogen for N2+lys-5 (i.e., overexpression of lys-5 in N2 background) and all strains on the non-pathogenic control. Under control conditions, individuals of almost all strains survived until the end of the assay period of seven days. Survival rates were analysed using the Kaplan-Meier approach followed by a post-hoc Log-rank test for comparisons between the KO mutants and the corresponding transgenic strain as well as for comparisons between the transgenic strains and N2. Significant differences between transgenic strains and the corresponding KO mutant are shown in bold and indicated by *. Significant differences between N2 and transgenic strains are indicated with +. Because of multiple testing we adjusted significance levels using FDR. (DOCX) [file pone.0024619.s004.docx]

**Table S4. Survival rate of lysozyme knock-out mutants and transgenic worms on pathogenic and non-pathogenic Bt.**

| **Strain** | **Pathogenic Bt** | **Non-pathogenic Bt** |
| --- | --- | --- |
| N2 wildtype | 3.59 (0.21) | 7 (0) |
| *lys-2(tm2398)* | 3.01 (0.19) | 7 (0) |
| *lys-5(tm2439)* | 2.39 (0.16) | 7 (0) |
| *lys-7(ok1386)* | 2.58 (0.14) | 6.8 (0.18) |
| N2 + *lys-2* | 3.42 (0.19) | 7 (0) |
| N2 + *lys-5* | **3.96 (0.22)*** | 7 (0) |
| N2 + *lys-7* | **5.12 (0.17)*^+^** | 7 (0) |
